# Micro-scale Experimental System Coupled with Fluorescence-based Estimation of Fungal Biomass to Study Utilisation of Plant Substrates

**DOI:** 10.1007/s00248-021-01794-9

**Published:** 2021-07-03

**Authors:** Julianna B. Németh, Dániel G. Knapp, Annamária Kósa, Panna Á. Hegedűs, Gábor Herczeg, Pál Vági, Gábor M. Kovács

**Affiliations:** 1 Department of Plant Anatomy, Institute of Biology, Eötvös Loránd University, Pázmány Péter sétány 1/C, Budapest, 1117 Hungary; 2 Behavioural Ecology Group, Department of Systematic Zoology and Ecology, Institute of Biology, Eötvös Loránd University, Pázmány Péter sétány 1/C, Budapest, 1117 Hungary

**Keywords:** Dark septate endophytes, Endophyte, Plant debris, Saprobes, Substrate utilisation

## Abstract

The degradation capacity and utilisation of complex plant substrates are crucial for the functioning of saprobic fungi and different plant symbionts with fundamental functions in ecosystems. Measuring the growth capacity and biomass of fungi on such systems is a challenging task. We established a new micro-scale experimental setup using substrates made of different plant species and organs as media for fungal growth. We adopted and tested a reliable and simple titration-based method for the estimation of total fungal biomass within the substrates using fluorescence-labelled lectin. We found that the relationship between fluorescence intensity and fungal dry weight was strong and linear but differed among fungi. The effect of the plant organ (i.e. root vs. shoot) used as substrate on fungal growth differed among plant species and between root endophytic fungal species. The novel microscale experimental system is useful for screening the utilisation of different substrates, which can provide insight into the ecological roles and functions of fungi. Furthermore, our fungal biomass estimation method has applications in various fields. As the estimation is based on the fungal cell wall, it measures the total cumulative biomass produced in a certain environment.

**Supplementary Information:**

The online version contains supplementary material available at 10.1007/s00248-021-01794-9.

## Introduction

The degradation capacity and utilisation of different complex plant substrates play crucial roles in the functioning of saprobic fungi as well as various plant symbionts with fundamental roles in ecosystems [1–3]. These features might contribute to *in planta* colonisation [4, 5] and to nutrient mobilisation from the soil in the case of mutualistic symbionts [6, 7]. Degradation capacities and preferences of the microbial community could be measured by substrate loss in situ using complex plant substrates, such as litter bags and microcontainers [8] or the tea bag method [9]. Microbial biomass on a certain substrate could also indicate substrate use, providing a basis for more precise estimations of utilisation capacities.

A wide range of methods have been developed to estimate the quantity of different microorganisms: counting and culturing microbes [10, 11], light microscopy using chemically cleared plant tissues combined with specific staining [12], measuring microorganism-derived contents or structures [13], colorimetric methods [14], microarray analyses [15], rRNA targeted fluorescence in situ hybridization (FISH) [16], FISH in combination with microautoradiography [17], isotope tracking [18], proteomic analyses [19], enzyme activity assays and numerous assays based on respiration or utilisation of different substrates [20, 21], and measuring the concentration of microbe-derived bioindicator molecules, such as phospholipid phosphate (PL-P) [22], ATP, and dehydrogenases [23]. Alternatively, PCR-based techniques [24, 25] such as RNA-or DNA-based qPCR, have been used for microbial quantification [26–29]. However, these approaches have their limitations [e.g. [30, 31], and they mostly detect live cells. These problems need to be addressed for fungal biomass estimation in industrial fields as well, as in the case of solid state fermentation (SSF) [32, 33].

In the soil or substrate, ergosterol, the most abundant sterol in the cell membrane of certain fungal groups [34], is rapidly degraded by oxidation and light. For this reason, ergosterol is a sensitive indicator, particularly for the estimation of living biomass [35]. Ergosterol content assays are accurate and reproducible; however, they are laborious and time-consuming [32]. Similarly, analyses of neutral lipid fatty acids (NLFAs) and phospholipid fatty acids (PLFAs) could help to reveal the physiological conditions of fungi [36] and could be used to quantify fungi, even *in planta* [37]; nevertheless, they also apply to the living biomass.

Glucosamine liberated from chitin by hydrolysis and deacetylation can also be used to estimate fungal biomass by using a staining and colorimetry method [38]. However, invertebrate chitin and the bacterial cell wall can also be sources of glucosamine or other hexosamines, making the exclusive detection of fungal-originated glucosamine impossible in field samples [39]. Unlike many other methods, chitin is an indicator of both living and dead fungal biomass due to its exceptional stability [40], and it is therefore a potential indicator of total biomass. Lectins, such as wheat germ agglutinin (WGA) conjugates, are widely used for optical imaging or even for the counting and measuring of fungal partners in different symbioses [40–46] due to the high affinity/specificity to chitin. WGA coupled with fluorescein has also been used for the fluorescence-based quantification of *in planta* colonisation of phytopathogenic fungi [45].

We aimed to develop an experimental setup in which different plant substrates are used to evaluate the growth capacity of fungi with respect to plant and tissue type. To test the experimental system and to illustrate its potential use, we applied it to an analysis of root endophytic fungi belonging to a widely distributed, phylogenetically diverse fungal group, expected to have wide variation in degradation capacity and preference for certain plant debris. These root-colonising endophytic fungi, called dark septate endophytes (DSEs) based on their septate and melanized hyphae [47], represent a diverse group of widespread and common fungi associated with plants in many ecosystems [48–51]. DSE fungi have been reported from numerous plant families [47, 52] and different DSE species are associated with distinct host plants or plant types (e.g. grasses, other herbaceous plants, or woody species) and habitats, such as grasslands or forests [52, 53]. Information about their function and ecological role is still limited [52, 54]. DSE fungi might have important functions as latent saprobes; however, only a few studies have focused on the saprobic features of species with strong saprobic ability and a broad spectrum of degrading enzymes [55–58]. Genomic analyses have also revealed the expansion of carbohydrate-active enzymes (CAZymes), especially plant cell wall-degrading enzymes (PCWDEs), in DSE fungi [59]. Although several differences and taxon-specific genomic features have been found, root endophytic fungi tend to have similar degradation enzyme repertoires [59]. These genomic features support the hypothesis that DSEs have an important ecological role due to their saprobic capacities. Their exclusive presence in roots and the variant relation to different hosts suggests a certain degree of tissue specificity [60, 61]. Although DSE fungi colonise roots and are occasionally soil saprobes, they also degrade senescent aboveground tissues. To resolve these issues, measurements of growth in controlled experimental setups and biomass estimation are of fundamental importance.

In this study, we developed a micro-scale experimental setup in which different plant substrates could be used as media for fungal growth. In these systems, we evaluated differences in the growth capacity of different DSE fungi with respect to plant and tissue type. Our aim was to measure the total biomass of the fungi that developed on certain plant materials. We adopted and developed a reliable, simple, quantitative WGA titration-based fluorimetric method to estimate the total fungal biomass within the substrates.

## Methods

### Plant substrates and fungal isolates

**Fig. 1:**
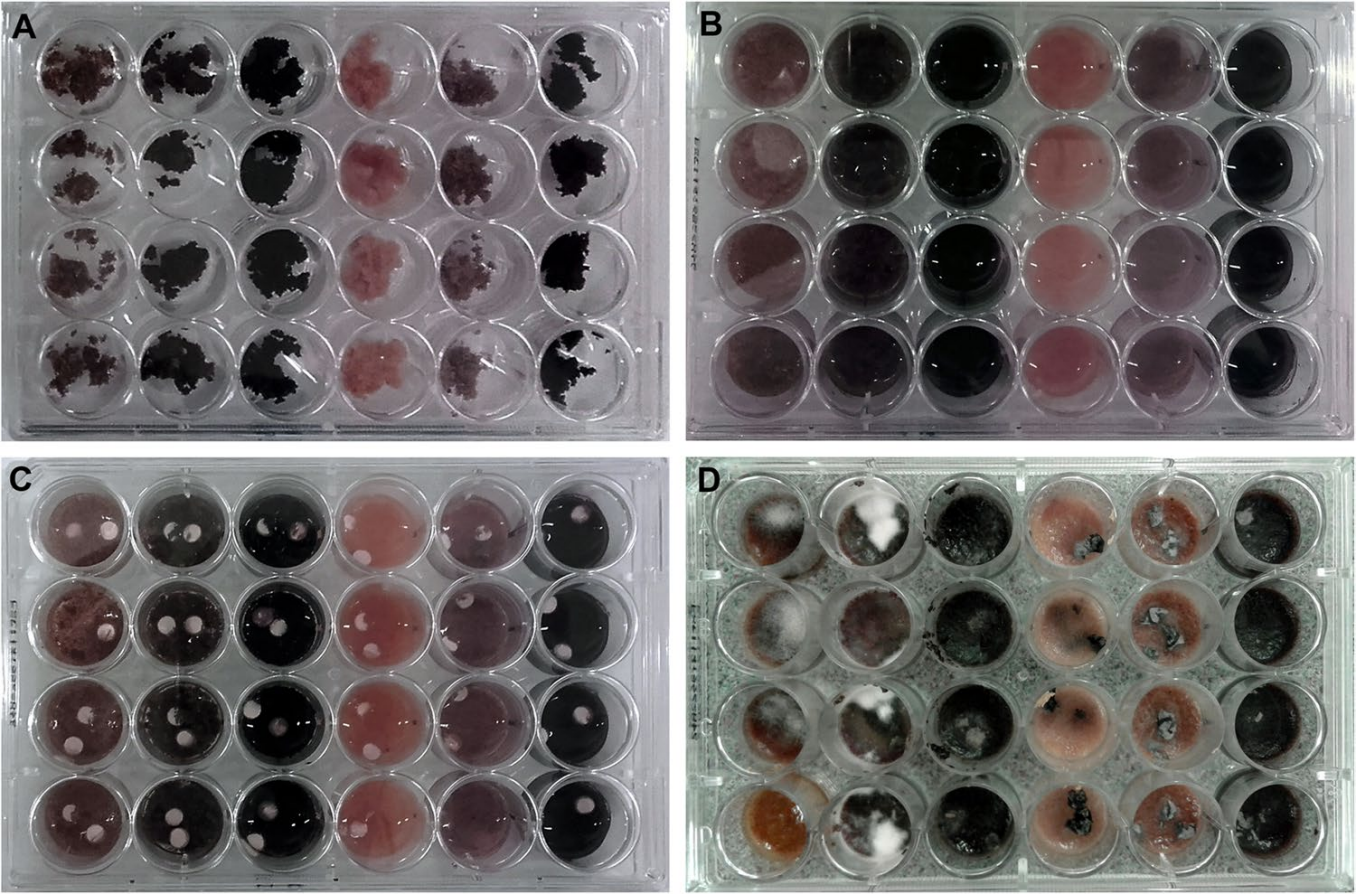
Experimental setup developed to test the growth capacity of different fungi on plant substrates. The 24-well tissue culture plates were filled with 6 different substrate types with four replicates (rows). The plate columns contained (from left to right): cabbage shoot, barley shoot, ribwort shoot, cabbage root, barley root, and ribwort root. (**A**) Wells containing plant materials (**B**) supplemented with 400 µL of sterile distilled water. (**C**) Substrates on the 24-well plate inoculated with two mycelium plugs of one isolate per well. (**d**) Plate with *Periconia macrospinosa* 2 weeks after inoculation

For the assessment of biomass, five DSE species were grown on different plant materials in 24-well tissue culture plates (Fig. 1). The shoot and root tissues of three plant species, barley (*Hordeum vulgare*, “Paris,” Agroszen Kft.), cabbage (*Brassica oleracea* var. *capitata*), and ribwort plantain (*Plantago lanceolata*). The plants represent phylogenetically distant groups, and as we aimed to screen the general saprobic capacities of endophytes, the original hosts of the fungi were not included. *Plantago* is a well-known mycorrhizal genus, while *Brassica* is a non-mycorrhizal plant. Barley is widely used in studies of fungal symbiosis [62, 63]. Seeds of each plant species were surface-sterilised in 70% ethanol for 5 min, rinsed 3 × in distilled water, rinsed in 30% H_2_O_2_ for 15 min, washed in distilled water five times, and germinated on water agar. After one week, seeds were planted in sterilised sandy soil (from the collection site of the fungal isolates as described in Knapp et al. [50]) mixed with zeolite in a 2:1 ratio. The plants were irrigated with sterilised tap water twice a week and with Hoagland solution [64] twice per month. Plants were harvested on week 9, and the root system and shoot of each species were ground separately in a mortar using liquid nitrogen. Then, the six substrate types were sterilised in an autoclave and stored at − 80 °C until further experiments. Autoclaving could influence the characteristics of the complex substrates; nevertheless, we wanted to avoid any kind microbial contamination in the experiments. With grinding, we could exclude the effects caused by inhomogeneous substrates.

The five DSE species used in the study (Table 1) represent taxonomically distant lineages with different host preferences and saprobic ability [50, 57]. *Periconia macrospinosa* and *Darksidea alpha* are widespread and dominant colonisers of grasses, *Setophoma terrestris*, *Cadophora* sp. (likely conspecific with the recently described *C. meredithiae* [65]), and the recently described *Polyphilus sieberi* [66] represent common endophytes associated with woody plant species (Table 1). The isolates were collected from the semiarid grasslands of Fülöpháza, Hungary [50]. Isolates were cultured for 3 weeks on modified Melin-Norkrans (MMN) agar [67] and for 2 weeks in case of fast-growing *P. macrospinosa*. The medium was covered with cellophane foil, enabling the easy removal of the mycelium, and 4-mm-diameter plugs were punched with a sterilised cork borer for inoculation.

**Table 1:** Fungal isolates used in the study

DSE species	Isolate no	Order	Host type
*Periconia macrospinosa*	DSE2036^a^	Pleosporales	Grass
*Setophoma terrestris*	REF109^b^	Pleosporales	Non-grass
*Darksidea alpha*	DSE-7/24^b,c^	Pleosporales	Grass
*Cadophora* sp.	DSE1049^a,d^	Helotiales	Non-grass
*Polyphilus sieberi*	REF052^e^	Helotiales	Non-grass

^a^For a detailed description of the strain, see Knapp et al. [59]; ^b^Strain introduced by Knapp et al. [50]; ^c^Strain and species information can be found in Knapp et al. [53]; ^d^Strain was introduced and examined in Vági et al. [68]; ^e^For a detailed description of the strain and the species, see Ashrafi et al.[66]

### Growth system

On each 24-well tissue culture plate, six different substrate types (barley shoot and root, cabbage shoot and root, and ribwort shoot and root) were added to one column, and four wells within the column were included as replicates of one fungal species on one specific substrate (Fig. 1), with one species per plate. The weight of the plant material added to each well was measured precisely (data not shown; the average weight was approximately 0.2 g/well). Then, 400 µL of sterile distilled water was added to each well and the wet substrate was inoculated with two fungal plugs per well (Fig. 1). The control and inoculated plates were incubated for 2 weeks at room temperature in the dark and kept frozen at − 80 °C until processing.

### Estimation of hyphal biomass

#### Physical extraction and fixation

The frozen samples were transferred to 5-mL plastic containers with 2 mL of distilled water. Samples were sonicated for 2 × 30 s using a Soniprep 150 Ultrasonic Disintegrator (MSE Ltd., London, UK) equipped with a 19-mm-diameter probe. After the sonication of each sample, the probe tip was rinsed into the container with another 1 mL of distilled water. Hence, the sonicated samples were diluted in 3 mL of distilled water.

The samples were then pipetted into 1.5-mL centrifuge tubes and spun down at 13,000 *g* for 5 min. The supernatant was discarded by pipetting, and the pellet was resuspended in 1 mL of Sörensen buffer (0.1 M, pH = 7.2). This washing procedure was repeated three times. After the last centrifugation step, the pellet was resuspended in 1 mL of Sörensen buffer.

#### Staining

After the washing steps, 100-µL aliquots of the suspension were transferred to 1.5-mL Eppendorf tubes and 400 µL of Sörensen buffer and 5 µL of 1 mg/mL WGA-AlexaFluor488 (Wheat Germ Agglutinin, Alexa Fluor 488 conjugate, Molecular Probes W11261; Thermo Fisher Scientific, Vilnius, Lithuania) were added to specifically stain the fungal material. Staining was performed overnight at 4 °C. To determine the amount of the WGA probe, the WGA concentration was increased until the fluorescence signal in the homogenates reached a maximum level (data not shown).

The stained suspensions were centrifuged at 13,000 *g* for 5 min. The supernatant was discarded, and the pellet was resuspended in 1 mL of Sörensen buffer. This washing step was repeated two additional times. Finally, the pellet was resuspended in 400 µL of a 50% glycerol/Sörensen solution to prevent sedimentation during fluorescence measurements.

#### Fluorescence measurements

Samples (400 µL) were added to glass tubes with an outer diameter of 6 mm and inner diameter of 4 mm, and the tubes were fixed sequentially in the cuvette holder of a Jobin–Yvon Horiba Fluoromax 3 spectrofluorometer (Paris, France). The linear range of the fluorescence, the measuring capacity and range of the instrument were tested, and samples were diluted accordingly. The excitation and emission wavelengths for WGA-AlexaFluor488 were determined on 1 mL of a 50% glycerol/Sörensen solution containing 1 µL of 1 mg/mL WGA-AlexaFluor488. The excitation wavelength was 493 nm (with a 2-nm slit width) and the emission maximum of the samples was 520 nm in the recorded range of 500–550 nm (with a 5-nm slit width). All measurements were made at 0.5-nm increments and 0.5-s integration times. For each sample, three spectra were automatically averaged using the software provided with the instrument. Fluorescence emission spectra were analysed using SPSERV V.11 (copyright Bagyinka, Cs., Biological Research Centre of HAS, Szeged, Hungary). Five-point linear smoothing and correction for the wavelength-dependent sensitivity of the photomultiplier were performed.

### Calibration of the hyphal biomass estimation

**Fig. 2:**
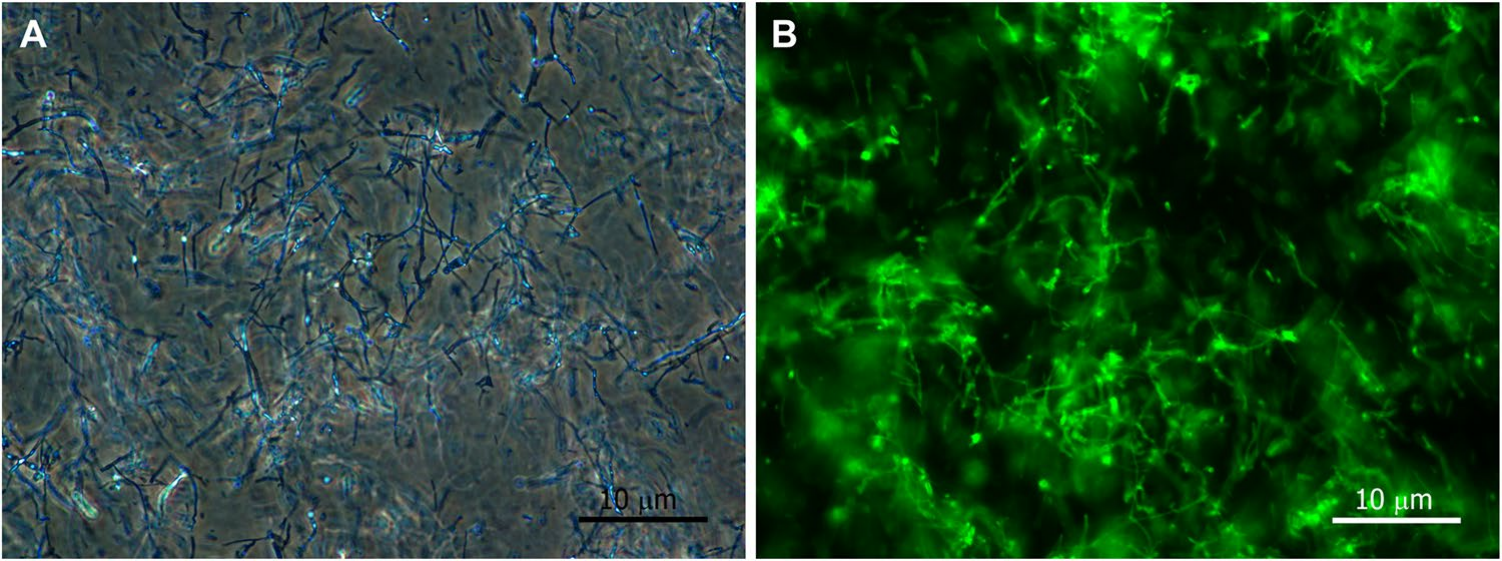
Micrographs of hyphal fragments. (**A**) Phase-contrast micrograph of unstained (for dry weight measurement) and (**B**) fluorescence image of WGA-AlexaFluor488-stained (for fluorescence measurement) hyphal fragments in sonicated *Periconia macrospinosa* liquid cultures. Bars: 10 µm

Our method was based on the assumption of a strong positive correlation between fungal biomass and the corresponding fluorescence intensity data. Experiments were carried out with all five fungi to test the direct correlation between fungal biomass and WGA-AlexaFluor488 emission intensity. The mycelium was collected from liquid cultures after 3 weeks and then washed in distilled water. Washing steps were followed by sonication of the fungal material in distilled water (Fig. 2). The sonicated suspension was divided into portions in previously weighed 1.5-mL centrifuge tubes, and four pairs of tubes were filled with aliquots of 100, 200, 300, or 400 µL of the suspension. One tube for each pair was supplemented with Sörensen buffer to 1 mL and was saturated overnight with WGA-AlexaFluor488. Then, they were washed as described above, and fluorescence emission spectra were recorded as previously described. The other tube of the pair was vacuum-dried to a constant weight in a vacuum concentrator (Univapo 100ECH, UniEquip, Munich, Germany), and the dried weight of each sample was recorded.

### Estimation of fungal biomass grown on different substrates

Substrates on non-inoculated control plates were measured as described above, and their emission spectra were subtracted from those of the corresponding stained and inoculated substrates. The biomass of certain species on different substrates was recorded as the fluorescence intensity in counts per second (cps) and was not converted to mass/weight.

### Statistical analyses

First, we were interested whether our new variable, fluorescence intensity, (i) described fungal dry weight in a linear manner with high explained variance and (ii) whether the relationship between fluorescence intensity and fungal dry weight was similar in different fungi. To this end, we fitted data from the fungal species in a General Linear Model (GLM) with fluorescence intensity as the dependent variable, fungal species, fungal dry weight and their interaction as fixed predictors. In this model, a significant fungal dry weight effect without a significant interaction term would have indicated a linear relationship with fluorescence intensity irrespective of fungal species, while a significant interaction would have indicated that the relationship is species-specific. This analysis was complemented with linear regressions between fluorescence intensity and fungal dry weight ran separately on the species.

Since the previous GLM yielded a highly significant interaction term with strong species-specific linear relationships (see Results), we could not analyse fluorescence intensity pooled across the fungal species. Therefore, we treated our fluorescence intensity measures in the four fungal species (in case of one species, the fluorescence intensities showed no correlation at all with the biomass; see Results) as different but non-independent variables that can be used to test the same hypothesis (i.e. effects of plant species and plant part). In this sense, we built a multivariate GLM with fluorescence intensity of the four fungal species as four response variables, plant species, plant part and their interaction as fixed effects. Linear regressions revealed that the weight of the plant material added to the wells never affected fluorescence in any of the fungal species (0.16 < P < 0.97) and thus the initial mass of substrate was not included in the main model. To interpret the multiGLM results, we first checked the multivariate results (Wilk’s λ) and only interpreted univariate (here: species-specific) F-tests when the given multivariate predictor was significant.

Because statistical significance (P value) alone is insufficient for the evaluation of the biological significance [69], we also provide effect sizes for the statistically significant effects in the form of partial η2 to provide an estimate of biological importance besides statistical significance. We interpreted partial η2 < ≈ 0.02 as small, partial η2 ≈ 0.13 as medium and partial η2 ≈ < 0.26 as large effects [70].

For the statistical analyses, the program package SPSS Version 24 was used.

## Results

All fungi used in the experiments grew well in the micro-scale system on different substrates. Since the fungi tested did not sporulate, the experimental setup (one fungus on one plate) was easy to manage with no cross-contamination between different substrates. No contamination in control wells was observed. The preparation of the fungal material for biomass measurements was relatively laborious; however, no hazardous chemical and/or method was necessary during the process. We summarized the methods applied in detailed protocols (Online Resource).

**Fig. 3:**
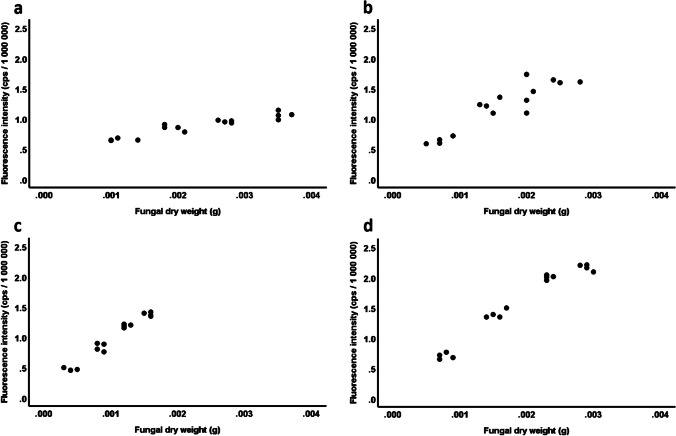
Species-specific linear fluorescence intensity–fungal dry weight relationships revealed by GLM based on dry hyphal weight (g) and average fluorescence (counts per second — cps) of hyphal suspensions of (**a**) *Periconia macrospinosa*, (**b**) *Cadophora* sp., (**c**) *Polyphilus sieberi,* and (**d**) *Darksidea alpha*

When the calibration measurements were carried the samples of *Setophoma terrestris* showed totally inconsequent fluorescence values, albeit we repeated the measurements several times. Because of that, albeit *S. terrestris* grown well in the experimental system, the data on this species were excluded from further analyses and worked with the data gained from the other four DSE fungi. A GLM revealed that the relationship between the fluorescence intensity and fungal dry weight was strongly divergent among fungal species (species: *F*_3,55_ = 3.65, P = 0.02; partial η^2^ = 0.17; dry weight: *F*_1,55_ = 549.38, P < 0.001; partial η^2^ = 0.91; species × dry weight: *F*_3,55_ = 53.84, P < 0.001; partial η^2^ = 0.75; Fig. 3). The linear regressions revealed that fungal dry weight accounted for more than 80% of variation in fluorescence intensity in the species, but the highly divergent slopes (B, unstandardized coefficient) depicted almost fivefold difference between species (*P. macrospinosa*: *R*^2^ = 0.88; B = 159.71; Standard Error of B [SE] = 15.68; *t* = 10.18; P < 0.001; *Cadophora* sp.: *R*^2^ = 0.83; B = 507.71; SE = 63.87; *t* = 7.95; P < 0.001; *P. sieberi*: *R*^2^ = 0.96; B = 781.21; SE = 41.48; *t* = 18.83; P < 0.001; *D. alpha*: *R*^2^ = 0.96; B = 701.53; SE = 40.00; *t* = 17.54; P < 0.001; Fig. 3). The relationship between fungal dry weight and fluorescence intensity was almost identical in *P. sieberi* and *D. alpha* but differed considerably in the other two species (Fig. 3).

**Fig. 4:**
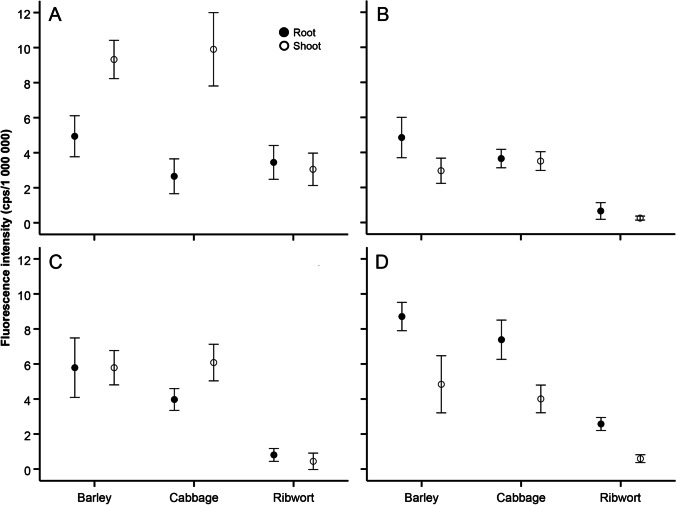
Effects of plant species (barley, cabbage, and ribwort) and plant part (root and shoot) on the development of the four DSE species determined by fluorescence intensity. The multi-GLM revealed a strong plant species × plant part interaction. Means + 95% confidence intervals are shown. (**A**) *Periconia macrospinosa*, (**B**) *Cadophora* sp., (**C**) *Polyphilus sieberi*, and (**D**) *Darksidea alpha*

Multivariate GLM revealed a strong multivariate effect of plant species × plant part interaction (plant species: Wilk’s λ = 0.02; *F*_8,28_ = 24.39; P < 0.001; partial η^2^ = 0.87; plant part: Wilk’s λ = 0.05; *F*_4,14_ = 66.91; P < 0.001; partial η^2^ = 0.95; plant species × plant part: Wilk’s λ = 0.05; *F*_8,28_ = 11.94; P < 0.001; partial η^2^ = 0.77). Subsequent univariate tests revealed that the interaction was significant and strong for all fungal species (5.10 < *F*_2,17_ < 40.81; all P < 0.018; 0.37 < η^2^ < 0.83; Fig. 4). Fungi tended to develop better on barley and cabbage than on ribwort (Fig. 4). The effect of plant part depended on both plant and fungal species; however, *P. macrospinosa* was a relative shoot-specialist, while *D. alpha* was a relative root-specialist compared with the utilisation patterns for the other two species (Fig. 4).

## Discussion

We established a microscale experimental setup, a novel small-scale SSF system, to test the growth capacities of different fungi on a range of plant substrates. The growing capacity of a certain fungus was not affected by the exact amount of plant material applied, showing that there was no striking inhomogeneity in the plant substrates used in the study. The WGA-based method coupled with quantitative fluorimetry biomass estimation was effective and could be used to estimate the total fungal growth in the substrates tested. Although the WGA affinity seems to result in taxon-specific fluorescence intensities with divergent slopes, fluorescence and fungal biomass showed a strong positive linear relationship. An initial test of the linearity of fluorescence intensity is obligatory because there are exceptional cases and species, such as *Setophoma terrestris*, where inconsequent results were gained and no correlation between the hyphal biomass and fluorescence could be detected.

Determination of the total fungal biomass in a substrate or in a living host is challenging. The method applied here is based on the specificity of WGA to the chitin content of the fungal cell wall [71] and confers strong contrast and, consequently, good sensitivity from the fluorescent label AlexaFluor488. Our method differed from the WGA-based fungal biomass estimation method developed and applied by Ayliffe et al. [45] for fungal disease progression in the leaves of crops. We tested the method on fungal species-specific systems using stained homogenates from cultures of fungi, instead of using purified crab shell particles as a chitin source [45]. Testing the WGA titration with all fungi involved in the experiment was crucial, not only because of the species-specific relationship between the fluorescence intensity and fungal dry weight we haveshown here, but also as there was a fungus (*S. terrestris*) that bound to WGA-AlexaFluor488, despite no correlation between binding and the fluorescent signal with fungal biomass. We applied AlexaFluor488 instead of fluorescein owing to its capacity for more stable signal generation by microscopy and its pH-insensitivity [72], making it an ideal candidate for fluorimetric analyses.

A comparison of the examined DSE fungi showed striking interspecific differences in growth capacity on different plant substrates. These differences might be due to differences in the metabolic, cell-wall, and tissue composition among plant species and organs, the amount of easily utilisable sources, and differences in the enzyme spectra of the fungi [57]. We found complementary features in the biomass profiles of different substrates of *P. macrospinosa* and *D. alpha*. Both are dominant grass-associated endophytes and may colonise the same root of the host. This might indicate a complementary functional repertoire, which is possibly important when colonising the same host. This functional complementarity fits within the concept of the plant holobiont [57, 73]. Interestingly, although all fungi tested were exclusively root-colonising soil fungi, only *D. alpha* showed better growth on root tissues compared to aboveground tissues. In general, ribwort root and shoot substrates result in lower fungal growth, probably due to various antimicrobial compounds that are typically produced by the herb [74].

Using six different complex substrates, our results revealed substrate-dependent differences in growth, suggesting differences in saprobic capacities of the root endophytes tested. This is consistent with differences in strategies, enzymatic toolboxes, and degrading capacities among DSEs in previous studies [55–57, 59].

In conclusion, a new reliable experimental system was developed to test the growth capacity and saprobic features in a small-scale solid fermentation system and to visualise and compare the biomass of different fungal species growing in plant material by quantitative fluorimetry based on the cell-wall specific AlexaFluor488-labelled WGA. This simple WGA-titration method could be adapted to large-scale SSF systems, soil research, investigations of symbiotic interactions, fungal pathogen studies in crop plants, livestock, or even human medicine.

## Supplementary Information

Below is the link to the electronic supplementary material.

Supplementary file1 (DOCX 25 KB)

## Data Availability

The datasets generated during and/or analyzed during the current study are available from the corresponding author on reasonable request.

## References

[1] Eastwood DC, Floudas D, Binder M, Majcherczyk A, Schneider P, Aerts A, Asiegbu FO, Baker SE, Barry K, Bendiksby M, Blumentritt M, Coutinho PM, Cullen D, de Vries RP, Gathman A, Goodell B, Henrissat B, Ihrmark K, Kauserud H, Kohler A, LaButti K, Lapidus A, Lavin JL, Lee YH, Lindquist E, Lilly W, Lucas S, Morin E, Murat C, Oguiza JA, Park J, Pisabarro AG, Riley R, Rosling A, Salamov A, Schmidt O, Schmutz J, Skrede I, Stenlid J, Wiebenga A, Xie X, Kües U, Hibbett DS, Hoffmeister D, Högberg N, Martin F, Grigoriev IV, Watkinson SC (2011). The plant cell wall-decomposing machinery underlies the functional diversity of forest fungi. Science.

[2] King BC, Waxman KD, Nenni NV, Walker LP, Bergstrom GC, Gibson DM (2011). Arsenal of plant cell wall degrading enzymes reflects host preference among plant pathogenic fungi. Biotechnol Biofuels.

[3] van den Brink J, de Vries RP (2011). Fungal enzyme sets for plant polysaccharide degradation. Appl Microbiol Biotechnol.

[4] Hacquard S, Kracher B, Hiruma K, Münch PC, Garrido-Oter R, Thon MR, Weimann A, Damm U, Dallery JF, Hainaut M, Henrissat B, Lespinet O, Sacristán S, Loren V, van Themaat E, Kemen E, McHardy AC, Schulze-Lefert P, O'Connell RJ (2016). Survival trade-offs in plant roots during colonization by closely related beneficial and pathogenic fungi. Nat Commun.

[5] Martin F, Kohler A, Murat C, Veneault-Fourrey C, Hibbett DS (2016). Unearthing the roots of ectomycorrhizal symbioses. NatRev Microbiol.

[6] Treseder K, Torn M, Masiello C (2006). An ecosystem-scale radiocarbon tracer to test use of litter carbon by ectomycorrhizal fungi. Soil Biol Biochem.

[7] Clemmensen KE, Bahr A, Ovaskainen O, Dahlberg A, Ekblad A, Wallander H (2013). Roots and associated fungi drive long-term carbon sequestration in boreal forest. Science.

[8] Eisenbeis G, Lenz R, Heiber T (1999). Organic residue decomposition: the minicontainer system—a multifunctional tool in decomposition studies. Environ Sci Pollut Res.

[9] Keuskamp JA, Dingemans BJJ, Lehtinen T, Sarneel JM, Hefting MM (2013). Tea Bag Index: a novel approach to collect uniform decomposition data across ecosystems. Methods Ecol Evol.

[10] Bloem J, Alef K, Nannipieri P (1995). Microscopic methods for counting bacteria and fungi in soil. Methods in applied microbiology and biochemistry.

[11] Baldrian P, Větrovský T, Cajthaml T, Dobiášová P, Petránková M, Šnajdr J, Eichlerová I (2013). Estimation of fungal biomass in forest litter and soil. Fungal Ecol.

[12] Phillips JM, Hayman DS (1970). Improved procedures for clearing roots and staining parasitic and vesicular-arbuscular mycorrhizal fungi for rapid assessment of infection. Trans Br Mycol Soc.

[13] Read DJ, Koucheki HK, Hodgson J (1976). Vesicular-arbuscular mycorrhiza in natural vegetation systems. I. The occurrence of infection. New Phytol.

[14] Hepper CM (1977). A colorimetric method for estimating vesicular-arbuscular mycorrhizal infection in roots. Soil Biol Biochem.

[15] Yergeau E, Schoondermark-Stolk SA, Brodie EL, Déjean S, DeSantis TZ, Gonçalves O, Piceno YM, Andersen GL, Kowalchuk GA (2009). Environmental microarray analyses of Antarctic soil microbial communities. ISME J.

[16] Bouvier T, Del Giorgio PA (2003). Factors influencing the detection of bacterial cells using fluorescence in situ hybridization (FISH): a quantitative review of published reports. FEMS Microbiol Ecol.

[17] Lee N, Nielsen PH, Andreasen KH, Juretschko S, Nielsen JL, Schleifer KH, Wagner M (1999). Combination of fluorescent in situ hybridization and microautoradiography—a new tool for structure-function analyses in microbial ecology. Appl Environ Microbiol.

[18] Haichar FEZ, Heulin T, Guyonnet JP, Achouak W (2016). Stable isotope probing of carbon flow in the plant holobiont. Curr Opin Biotechnol.

[19] Keiblinger KM, Wilhartitz IC, Schneider T, Roschitzki B, Schmid E, Eberl L, Riedel K, Zechmeister-Boltenstern S (2012). Soil metaproteomics — comparative evaluation of protein extraction protocols. Soil Biol Biochem.

[20] Pritsch K, Courty PE, Churin JL, Cloutier-Hurteau B, Ali MA, Damon C, Duchemin M, Egli S, Ernst J, Fraissinet-Tachet L, Kuhar F, Legname E, Marmeisse R, Müller A, Nikolova P, Peter M, Plassard C, Richard F, Schloter M, Selosse M-A, Franc A, Garbaye J (2011). Optimized assay and storage conditions for enzyme activity profiling of ectomycorrhizae. Mycorrhiza.

[21] Blagodatskaya E, Blagodatsky S, Anderson TH, Kuzyakov Y (2014). Microbial growth and carbon use efficiency in the rhizosphere and root-free soil. PLoS ONE.

[22] Hill TCJ, McPherson EF, Harris JA, Birch P (1993). Microbial biomass estimated by phospholipid phosphate in soils with diverse microbial communities. Soil Biol Biochem.

[23] Steudler S, Bley T (2015). Better one-eyed than blind—challenges and opportunities of biomass measurement during solid-state fermentation of basidiomycetes. Adv Biochem Eng Biotechnol.

[24] Tellenbach C, Grunig CR, Sieber TN (2010). Suitability of quantitative real-time pcr to estimate the biomass of fungal root endophytes. Appl Environ Microbiol.

[25] Baldrian P, Kolařík M, Štursová M, Kopecký J, Valášková V, Větrovský T, Zifčáková L, Snajdr J, Rídl J, Vlček C, Voříšková J (2012). Active and total microbial communities in forest soil are largely different and highly stratified during decomposition. ISME J.

[26] Nicholson P, Simpson D, Weston G, Rezanoor H, Lees A, Parry D, Joyce D (1998). Detection and quantification of *Fusarium culmorum* and *Fusarium graminearum* in cereals using PCR assays. Physiol Mol Plant Pathol.

[27] Silvar C, Díaz J, Merino F (2005). Real-time polymerase chain reaction quantification of *Phytophthora capsici* in different pepper genotypes. Phytopathology.

[28] Larsen RC, Vandemark GJ, Hughes TJ, Grau CR (2007). Development of a real-time polymerase chain reaction assay for quantifying *Verticillium albo-atrum* DNA in resistant and susceptible alfalfa. Phytopathology.

[29] Klosterman SJ (2012) Real-time PCR for the quantification of fungi in planta. Humana Press, 121–132.10.1007/978-1-61779-501-5_8 10.1007/978-1-61779-501-5_8 22183651

[30] Joergensen R, Wichern F (2008). Quantitative assessment of the fungal contribution to microbial tissue in soil. Soil Biol Biochem.

[31] Kralik P, Ricchi M (2017). A basic guide to real time PCR in microbial diagnostics: definitions, parameters, and everything. Front Microbiol.

[32] Desgranges C, Vergoignan C, Georges M, Durand A (1991). Biomass estimation in solid state fermentation I. Manual biochemical methods. Appl Microbiol Biotechnol.

[33] Singhania RR, Patel AK, Soccol CR, Pandey A (2009). Recent advances in solid-state fermentation. Biochem Eng J.

[34] Weete JD, Abril M, Blackwell M (2010). Phylogenetic distribution of fungal sterols. PLoS ONE.

[35] Gessner M, Newell SY, Christon HJ (2002). Biomass, growth rate, and production of filamentous fungi in plant litter. Manual of environmental microbiology.

[36] Baath E (2003). The use of neutral lipid fatty acids to indicate the physiological conditions of soil fungi. Microb Ecol.

[37] Vestberg M, Palojarvi A, Pitkansen T, Kaipainen S, Puolakka E, Keskitalo M (2012). Neutral lipid fatty acid analysis is a sensitive marker for quantitative estimation of arbuscular mycorrhizal fungi in agricultural soil with crops of different mycotrophy. Agric Food Sci.

[38] Ride JP, Drysdale RB (1972). A rapid method for the chemical estimation of filamentous fungi in plant tissue. Physiol Plant Pathol.

[39] Matcham SE, Jordan BR, Wood DA (1985). Estimation of fungal biomass in a solid substrate by three independent methods. Appl Microbiol Biotechnol.

[40] Nilsson K, Bjurman J (1998). Chitin as an indicator of the biomass of two wood-decay fungi in relation to temperature, incubation time, and media composition. Can J Microbiol.

[41] O’Connell RJ, Ride JP (1990). Chemical detection and ultrastructural localization of chitin in cell walls of *Colletotrichum lindemuthianum*. Physiol Mol Plant Pathol.

[42] Pain NA, Green JR, Gammie F, O’connell RJ,  (1994). Immunomagnetic isolation of viable intracellular hyphae of *Colletotrichum lindemuthianum* (Sacc. & Magn.) Briosi & Cav. from infected bean leaves using a monoclonal antibody. New Phytol.

[43] Perfect SE, Pixton KL, O’Connell RJ, Green JR (2000). The distribution and expression of a biotrophy-related gene, CIH1, within the genus *Colletotrichum*. Mol Plant Pathol.

[44] Andrade-Linares DR, Grosch R, Franken P, Rexer KH, Kost G, Restrepo S, Cepero C, de Garcia M, Maximova E (2011). Colonization of roots of cultivated *Solanum lycopersicum* by dark septate and other Ascomycetous endophytes. Mycologia.

[45] Ayliffe M, Periyannan SK, Feechan A, Dry I, Schumann U, Wang M-B, Pryor A, Lagudah E (2013). A simple method for comparing fungal biomass in infected plant tissues. Mol Plant Microbe Interact.

[46] Knapp DG, Imrefi I, Boldpurev E, Csíkos S, Berek-Nagy PJ, Akhmetova G, Otgonsuren B, Kovács GM (2019). Root colonizing endophytic fungi of the dominant grass *Stipa krylovii* from a Mongolian steppe grassland. Front Microbiol.

[47] Jumpponen A, Trappe JM (1998). Dark septate endophytes : a review of facultative biotrophic root-colonizing fungi. New Phytol.

[48] Mandyam K, Jumpponen A (2005). Seeking the elusive function of the root-colonising dark septate endophytic fungi. Stud Mycol.

[49] Porras-Alfaro A, Herrera J, Sinsabaugh RL, Odenbach KJ, Lowrey T, Natvig DO (2008). Novel root fungal consortium associated with a dominant desert grass. Appl Environ Microbiol.

[50] Knapp DG, Pintye A, Kovács GM (2012). The dark side is not fastidious – dark septate endophytic fungi of native and invasive plants of semiarid sandy areas. PLoS ONE.

[51] Qin Y, Pan X, Kubicek C, Druzhinina I, Chenthamara K, Labbé J, Yuan Z (2017). Diverse plant-associated pleosporalean fungi from saline areas: ecological tolerance and nitrogen-status dependent effects on plant growth. Front Microbiol.

[52] Sieber TN, Grünig CR, Eshel A, Beeckman T (2013). Fungal root endophytes. Plant roots: the hidden half.

[53] Knapp DG, Kovács GM, Zajta E, Groenewald JZ, Crous PW (2015). Dark septate endophytic pleosporalean genera from semiarid areas. Persoonia.

[54] Porras-Alfaro A, Bayman P (2011). Hidden fungi, emergent properties: endophytes and microbiomes. Annu Rev Phytopathol.

[55] Caldwell BA, Jumpponen A, Trappe JM (2000). Utilization of major detrital substrates by dark-septate, root endophytes. Mycologia.

[56] Mandyam K, Loughin T, Jumpponen A (2010). Isolation and morphological and metabolic characterization of common endophytes in annually burned tallgrass prairie. Mycologia.

[57] Knapp DG, Kovács GM (2016). Interspecific metabolic diversity of root-colonizing endophytic fungi revealed by enzyme activity tests. FEMS Microbiol Ecol.

[58] Lacercat-Didier L, Berthelot C, Foulon J, Errard A, Martino E, Chalot M, Blaudez D (2016). New mutualistic fungal endophytes isolated from poplar roots display high metal tolerance. Mycorrhiza.

[59] Knapp DG, Németh JB, Barry K, Hainaut M, Henrissat B, Johnson J, Kuo A, Lim JHP, Lipzen A, Nolan M, Ohm RA, Tamás L, Grigoriev IV, Spatafora JW, Nagy LG, Kovács GM (2018). Comparative genomics provides insights into the lifestyle and reveals functional heterogeneity of dark septate endophytic fungi. Sci Rep.

[60] Rodriguez RJ, White JF, Arnold AE, Redman RS (2009). Fungal endophytes: diversity and functional roles. New Phytol.

[61] Wearn JA, Sutton BC, Morley NJ, Gange AC (2012). Species and organ specificity of fungal endophytes in herbaceous grassland plants. J Ecol.

[62] Grace EJ, Cotsaftis O, Tester M, Smith FA, Smith SE (2009). Arbuscular mycorrhizal inhibition of growth in barley cannot be attributed to extent of colonization, fungal phosphorus uptake or effects on expression of plant phosphate transporter genes. New Phytol.

[63] Lahrmann U, Ding Y, Banhara A, Rath M, Hajirezaei MR, Döhlemann S (2013). Host-related metabolic cues affect colonization strategies of a root endophyte. Proc Natl Acad Sci U S A.

[64] Hoagland DR, Broyer TC (1936). General nature of the process of salt accumulation by roots with description of experimental methods. Plant Physiol.

[65] Walsh E, Duan W, Mehdi M, Naphri K, Khiste S, Scalera A, Zhang N (2018). *Cadophora meredithiae* and *C. interclivum*, new species from roots of sedge and spruce in a western Canada subalpine forest. Mycologia.

[66] Ashrafi S, Knapp DG, Blaudez D, Chalot M, Maciá-Vicente JG, Zagyva I, Dababat AA, Maier W, Kovács GM (2018). Inhabiting plant roots, nematodes, and truffles—*Polyphilus*, a new helotialean genus with two globally distributed species. Mycologia.

[67] Marx DH (1969). The influence of ectotrophic mycorrhizal fungi on the resistance of pine roots to pathogenic infections. II. Production, identification, and biological activity of antibiotics produced by *Leucopaxillus cerealis* var. *piceina*. Phytopathology.

[68] Vági P, Knapp DG, Kósa A, Seress D, Horváth ÁN, Kovács GM (2014). Simultaneous specific *in planta* visualization of root-colonizing fungi using fluorescence *in situ* hybridization (FISH). Mycorrhiza.

[69] Nuzzo R (2014). Scientific Method: Statistical Errors. Nature.

[70] Cohen J (1988). Statistical Power Analysis for the Behavioral Sciences.

[71] Meyberg M (1988). Selective staining of fungal hyphae in parasitic and symbiotic plant-fungus associations. Histochemistry.

[72] Panchuk-Voloshina N, Haugland RP, Bishop-Stewart J, Bhalgat MK, Millard PJ (1999). Alexa dyes, a series of new fluorescent dyes that yield exceptionally bright, photostable conjugates. J Histochem Cytochem.

[73] Vandenkoornhuyse P, Quaiser A, Duhamel M, Le Van A, Dufresne A (2015). The importance of the microbiome of the plant holobiont. New Phytol.

[74] Nostro A, Germano MP, D’Angelo V, Marino A, Cannatelli MA (2000). Extraction methods and bioautography for evaluation of medicinal plant antimicrobial activity. Lett Appl Microbiol.

