# Supplementary material for: Micro-scale Experimental System Coupled with Fluorescence-based Estimation of Fungal Biomass to Study Utilisation of Plant Substrates
Source: Microb Ecol. 2021 Jul 3;83(3):714–23. doi: 10.1007/s00248-021-01794-9 (PMC8979871; doi:10.1007/s00248-021-01794-9)
Supplement: Supplementary file 1 — Supplementary file1 (DOCX 25 KB) [file 248_2021_1794_MOESM1_ESM.docx]

**Protocols**

**Preparation of the micro-scale experimental system**

1. The seeds of the plants were sterilised in 70% EtOH and washed in distilled water for 3 × 3 min.
2. The seeds were further sterilised in 30% H_2_O_2_ and washed in distilled water for 5 × 3 min.

**Note#** This worked with barley, cabbage, and ribwort but should be tested for other taxa.

1. Germinate the seeds on 1.5% water agar in Petri dishes. (Phytagel could also work; we found that paper (either paper towel or filter paper) was more complicated.)
2. After one week, plant seedlings in sterilised soil: zeolite 2:1, and grow for 1–2 months (depending on the species).
3. Harvest the plants and wash roots thoroughly.
4. Separate organs, if needed for the experiment.
5. Ground the plant tissues of each species separately in mortar in liquid nitrogen.
6. Sterilise the homogenous plant substrates in an autoclave.
7. Store at -80°C until utilization.
8. Use a sterile 24-well tissue culture plate.
9. Design the experiment to use a single fungal taxon per plate to avoid cross-contamination.
10. Always apply controls with no inoculation, preferably on separate plate(s) to avoid contamination.
11. Work under sterile conditions.
12. Add an approximately equal volume/mass of the homogenous plant substrate types to the wells in replicates on the same plate, avoiding contamination.

Ensuring identical weights might be highly laborious and complicated; therefore, we added approximately the same amount (in our case, ~0.2 g) and measured the plate after filling the wells. By this approach, we obtained the exact weight for all wells and tested the effect of the amounts in the statistical analyses; no effect (in our case) was observed, for example, on the homogeneity of the substrate and the mass-independent growth of fungi.

1. Wet the substrate with sterile distilled water (in our case, 400 µL) before fungal inoculation.

**Fungal inoculation**

1. Prepare plates with the appropriate medium and cover with cellophane.
2. Put a plug of the fungal strain in the centre of the plate.

**Note#** The growth rate of the fungus to be tested should be known. For replicates, sufficient mycelial plugs were obtained from the same radial distances.

1. Grow isolates for the appropriate duration.
2. Remove the mycelium from the cellophane and cut plugs with a sterilised cork borer from the same radial region of a strain.
3. Add two fungal plugs to each well to inoculate the substrates.
4. Cover the plate with a lid and seal with Parafilm, leaving minor slits.
5. Incubate the plates for 2 weeks in the dark at a the appropriate temperature (the length can differ depending on the growth rate).
6. After incubation, keep samples frozen at -80°C until further processing.

**Calibration of the biomass estimation**

Calibration for each fungal species is necessary to account for taxon-specific fluorescence intensities/reactions.

1. For calibration, grow each fungus in liquid medium or on solid medium, as described for the fungal inoculum preparation.
2. Collect 0.05–0.1 g of actively growing mycelium of each fungus into Eppendorf tubes
3. If liquid medium was used, centrifuge the suspension at 13000 × *g* for 5 min and discard the supernatant.
4. Add 2 mL of distilled water, suspend the pellet, centrifuge, and repeat the washing step once more.
5. Transfer the washed mycelium in 2 mL of distilled water to plastic containers (e.g. 5–10 mL tubes or vials).
6. Sonicate the samples for 2 × 30 s using an ultrasonic disintegrator and rinse the sonicator tip with another 1 mL of distilled water.
7. Pipette 1.5 mL of the sonicated samples into an Eppendorf tube centrifuge at 13000 × *g* for 5 min.
8. Discard the supernatant.
9. Pipette the remaining 1.5 mL of the sonicated samples into the Eppendorf tube and centrifuge at 13000 × *g* for 5 min.
10. Discard the supernatant.
11. Add 1 mL of Sörensen buffer (0.1 M, pH = 7.2), suspend the pellet in the buffer, and centrifuge for 5 min. Discard the supernatant.
12. Repeat the washing procedure two additional times.
13. After the last washing step, suspend the pellet in 2 mL of Sörensen buffer, transfer samples to plastic containers (e.g. 20–50 mL tubes or vials), and add 8 mL of Sörensen buffer to a 10-mL suspension.
14. After vigorous vortexing, split the suspension into portions immediately; transfer four replicates of 200, 400, 600, and 800 µL aliquots of the suspension to Eppendorf tubes (vortexing between transfer steps could be applied).
15. Then, halve each of the aliquots and transfer one half to another pre-weighed 1.5-mL Eppendorf tube.
16. Pairs of suspensions are obtained for (i) mass and (ii) fluorescence measurements from 100, 200, 300, and 400 µL.
17. *Weight:* Dry the suspensions in weighed Eppendorf tubes in a vacuum concentrator, determine the weight of each sample, and calculate the mycelial weight.
18. *Fluorescence measurement:* Supplement aliquots with an appropriate volume of Sörensen buffer to obtain final suspensions of 500 µL in each tube.
19. Add 5 µL of 1 mg/mL WGA-AlexaFluor488 solution to each tube.
20. Incubate the tubes overnight in the dark at 4°C
21. Centrifuge the stained suspensions at 13000 × *g* for 5 min.
22. Discard the supernatant, add 1 mL of Sörensen buffer (0.1 M, pH = 7.2) and suspend the pellet.
23. Repeat this washing procedure two additional times.
24. After the last centrifugation step, suspend the pellet in 400 µL of a 50% glycerol/Sörensen solution to prevent sedimentation during fluorescence measurements.
25. For fluorescent measurements, add appropriate sample volumes (according to the type and size of the cuvette holder of the spectrofluorometer). Always mix the samples before obtaining appropriate volumes.

***Note#*** The linear range of the fluorescence, the measuring capacity and range of the instrument should be tested, and samples should be diluted accordingly.

1. Set the excitation wavelength to 493 nm (with a 2-nm slit width) and emission maximum to 520 nm in the recorded range of 500–550 nm (with a 5-nm slit width) for WGA-AlexaFluor488.

**Note#** Settings depend on the fluorophore attached to WGA.

1. Determine the measured values (using 0.5 nm increments, 0.5 s integration times and correction for the wavelength-dependent sensitivity of the photomultiplier) for 1 mL of 50% glycerol/Sörensen solution containing 1 µL of 1 mg/mL WGA-AlexaFluor488.

*These measures indicate the biomass and the corresponding fluorescence intensities for test materials if there is a correlation between fungal biomass and fluorescence intensities.*

**Measuring fluorescence using the microscale experimental setup**

1. Transfer the frozen colonised (or control) substrates from the 24-well tissue culture plates to plastic containers (e.g. 5–10 mL tubes or vials) and supplement with 2 mL of distilled water.
2. Sonicate the samples for 2 × 30 s using an ultrasonic disintegrator and rinse the sonicator tip with another 1 mL of distilled water
3. Pipette 1.5 mL of the sonicated samples into an Eppendorf tube and centrifuge at 13000 × *g* for 5 min.
4. Discard the supernatant.
5. Pipette the remaining 1.5 mL of the sonicated samples into the Eppendorf tube and centrifuge at 13000 × *g* for 5 min.
6. Discard the supernatant.
7. Add 1 mL of Sörensen buffer (0.1 M, pH = 7.2), suspend the pellet in the buffer, and centrifuge for 5 min. Discard the supernatant.
8. Repeat this washing procedure two additional times.
9. After the last washing step, suspend the pellet in 2 mL of Sörensen buffer.
10. To stain hyphal particles, transfer 100 µL of the suspension immediately after vigorous vortexing to a new 1.5-mL Eppendorf tube.
11. Add 400 µL of Sörensen buffer
12. Add 5 µL of 1 mg/mL WGA-AlexaFluor488 solution.
13. Incubate the tubes overnight in the dark at 4°C.
14. Centrifuge the stained suspensions at 13000 × *g* for 5 min.
15. Discard the supernatant, add 1 mL of Sörensen buffer (0.1 M, pH = 7.2), and suspend the pellet.
16. Repeat this washing procedure two additional times.
17. After the last centrifugation step, suspend the pellet in 400 µL of a 50% glycerol/Sörensen solution to prevent sedimentation during fluorescence measurements.
18. For fluorescent measurements, add appropriate sample volumes (according to the type and size of the cuvette holder of the spectrofluorometer). Always mix the samples before samples are taken.

***Note#*** The linear range of the fluorescence, the measuring capacity and range of the instrument should be tested, and samples should be diluted accordingly.

1. For fluorescent measurements, appropriate volumes (according to the type and size of the cuvette holder of the spectrofluorometer) of the 400 µL samples. Care is taken to always mix the samples before appropriate volumes are taken.
2. The excitation wavelength was set to 493 nm (with 2 nm slit width) and emission maximum of the samples was set to 520 nm in the recorded range of 500–550 nm (with a 5 nm slit width) for the WGA-AlexaFluor488.

**Note#** The parameter settings depend on the fluorophore attached to WGA

1. Determine the measured values (using 0.5 nm increments, 0.5 s integration times and correction for the wavelength-dependent sensitivity of the photomultiplier) using 1 mL of a 50% glycerol/Sörensen solution containing 1 µL of 1 mg/mL WGA-AlexaFluor488.

**Additional note#**

Plant materials could have autofluorescence, which might influence fluorophore measurements. Evaluating the specific plant material might be useful; although we have found that AlexaFluor488 is a generally good stain (Vági et al., 2014), some plant materials may fully mask the stain.

Vági, P., Knapp, D. G., Kósa, A., Seress, D., Horváth, Á. N., & Kovács, G. M. (2014). Simultaneous specific *in planta* visualization of root-colonizing fungi using fluorescence *in situ* hybridization (FISH). Mycorrhiza, 24, 259–266. https://doi.org/10.1007/s00572-013-0533-8
